# The Association of Helicobacter pylori With Portal Hypertensive Gastropathy in Patients With and Without Cirrhosis

**DOI:** 10.7759/cureus.31183

**Published:** 2022-11-07

**Authors:** . Noor, Hala Mansoor, Rafi Ud Din, Muhammad Asif Farooq, Asma Asghar, Muhammad Siddique

**Affiliations:** 1 Gastroenterology and Hepatology, Combined Military Hospital (CMH) Lahore Medical College and Institute of Dentistry/National University of Medical Sciences (NUMS), Lahore, PAK; 2 Gastroenterology and Hepatology, Combined Military Hospital (CMH) Lahore, Lahore, PAK; 3 Gastroenterology and Hepatology, Combined Military Hospital (CMH) Kharian, Kharian, PAK

**Keywords:** non-cirrhotic, liver disease, cirrhosis, portal hypertensive gastropathy (phg), helicobacter pylori

## Abstract

Introduction: Cirrhosis and its associated complication of portal hypertensive gastropathy (PHG), among others, remain a significant cause of death in resource-poor countries with limited capacity for liver transplantation. This research aimed to assess the association of *Helicobacter pylori* (*H. pylori*) with portal hypertensive gastropathy and its severity in patients with and without cirrhosis.

Methodology: The study was conducted at a tertiary care hospital in Pakistan from April 2021 to May 2022. Liver cirrhosis was diagnosed by clinical manifestations, ultrasonography, and laboratory investigations. The severity of liver cirrhosis was assessed using the Child-Pugh scoring system. The association of *H. pylori* with portal hypertensive gastropathy in patients with and without cirrhosis was assessed using the chi-square test.

Results: A total of 120 patients participated in the study, of which 40 were without liver cirrhosis, while 80 were with cirrhosis. Among patients with cirrhosis, 24 were in Child-Pugh class A, 26 in class B, and 30 in class C. Of patients with liver cirrhosis who were *H. pylori*-negative, 37.5% (15/40) had portal hypertensive gastropathy. Of these, 12.5% (5/40) had severe PHG, while 25% (10/40) had mild PHG. Of patients with liver cirrhosis who were *H. pylori-*positive, 62.5% (25/40) had PHG. Of these, 2.5% (1/40) had severe PHG, while 60% (24/40) had mild PHG. *Helicobacter pylori* contributed nonsignificantly (p=0.080), showing no association with portal hypertensive gastropathy.

Conclusion: *Helicobacter pylori* does not appear to have any significant association to cause or worsen portal hypertensive gastropathy in patients with liver cirrhosis.

## Introduction

Liver cirrhosis results in scarring and damage. Scar tissue replaces the healthy tissue and prevents the liver from functioning normally. Around the globe, liver cirrhosis is a serious health concern, particularly in developing and underdeveloped countries with a high prevalence of hepatitis B and C. It may cause changes in gastric mucosa, termed portal hypertensive gastropathy (PHG), which can lead to potentially serious upper gastrointestinal (GI) bleeding [1].

Portal hypertensive gastropathy is characterized by the presence of an erythematous, mosaic-like appearance of the gastric mucosa with or without red spots described as snakeskin with a potential to bleed. It leads to compromised mucosal defense [2]. The prevalence of PHG ranges from 4% to 98% in different studies and is higher in those studies that included patients with advanced liver disease. Numerous pathophysiological mechanisms have been proposed for PHG, including gastric blood flow changes leading to diminished integrity of the epithelium, which causes hypoxia of the mucosal surface and increasing level of nitric oxide levels, the existence of *Helicobacter pylori* (*H. pylori*) infection, and decreased level of prostaglandin in the gastric mucosa [2].

*Helicobacter pylori* is a bacterial infection of the digestive system with high prevalence in developing countries where it is a key public health concern. Only in 2015, about 4.4 billion people tested positive for* H. pylori* globally [3]. *Helicobacter pylori* causes the release of various pro-inflammatory cytokines, including tumor necrosis factor alpha. It can cause iron deficiency anemia, gastroduodenal erosions, or ulceration, which can bleed massively. One of the dreaded complications of *H. pylori* infection is gastric cancer, especially adenocarcinoma and mucosa-associated lymphoid tissue (MALT) lymphomas. However, there is a controversy regarding its relationship with PHG in patients with liver cirrhosis [4-7]. Understanding the frequency of *H. pylori* infection in patients with liver cirrhosis and its association with PHG can be helpful in comprehending its probable involvement [3]. Theoretically, if *H. pylori* infection contributes to PHG pathogenesis, then its eradication would be helpful in its management, thus avoiding or reducing the potential complications of anemia and bleeding.

This study aimed to assess the association of *H. pylori* infection with PHG and its severity in patients with and without cirrhosis.

## Materials and methods

This study was conducted at the Combined Military Hospital Lahore from April 2021 to May 2022 after obtaining approval from the research ethics committee, in accordance with the principles of the Declaration of Helsinki. A minimum sample size of 100 patients was calculated using the Australian Bureau of Statistics online sample size calculator, assuming a 95% confidence interval and 5% margin of error, using the prevalence of portal hypertensive gastropathy in 51% of patients with liver cirrhosis in the study by Gjeorgjievski and Cappell as a reference [1].

The study included 120 patients, which were selected by non-probability consecutive sampling technique, out of which 80 patients were with liver cirrhosis, while 40 were without it. The inclusion criteria of our study comprised patients with and without cirrhosis who gave informed consent and were adults >18 years of age. Cirrhosis could be due to any cause, such as hepatitis B and/or C, and alcohol and nonalcoholic steatohepatitis (NASH). The patients included in the study were from inpatient medical wards, gastroenterology outpatient clinics, and the endoscopy suite.

The following patients were excluded from the study: all pregnant and lactating females, patients under 18 years of age, those with hepatocellular carcinoma (HCC) or any other malignancy, those with a history of surgical procedures for correction of portal hypertension or any other gastric surgeries, patients with portal, hepatic, or splenic thrombosis, those with active peptic ulcer, those with previous band ligation or cyanoacrylate injection, those on nonsteroidal anti-inflammatory drugs or proton pump inhibitors, those with a recent intake of antibiotics within the previous one month or *H. pylori* eradication therapy, those not willing to participate, and those with systemic diseases affecting the gastrointestinal tract.

Cirrhosis was diagnosed by clinical manifestations such as jaundice, palmar erythema, spider naevi, ascites, and splenomegaly, as well as by specific radiological and laboratory investigations. Laboratory investigations included fasting blood sugar, complete blood count, serum sodium (Na), bilirubin (Bil), alanine aminotransferase (ALT), alkaline phosphatase (ALP), albumin (Alb), creatinine (Creat), prothrombin time (PT), international normalized ratio (INR), hepatitis profile of B and C, and stool for *H. pylori* antigen.

Ultrasonography of the abdomen was done using LOGIQ (GE Healthcare, Chicago, IL, USA) in addition to a convex probe of 3.75 megahertz. It covered the assessment of the liver (focal lesion, portal vein size, and pattern of echo), the assessment of the spleen (pattern of echo and size), and the existence of ascites. All patients underwent upper GI endoscopy. The classification of esophageal varices was done using the modified Paquet classification as grade I (extends beyond the mucosal level), grade II (occupies one-third of the esophageal diameter, which is not compressed with air insufflation), and grade III (occupies 50% of the luminal diameter, in which varices are in contact with each other). PHG was recorded as per the grading system of Baveno III as either mild or severe. In mild PHG, a pink mucosal pattern is noticed without red signs or without the presence of black and/or brown spots. In severe PHG, a red mucosal pattern with red or brown spots is seen [4].

The severity of liver cirrhosis was assessed using the Child-Pugh score (Table 1). This classification breaks down patients into three classes. The Child-Pugh score of 5-6 points is class A, 7-9 points is class B, and 10-15 points is class C. The parameters, which are assessed to calculate these scores, include the degree of ascites, serum concentrations of bilirubin and albumin, prothrombin time, and the degree of encephalopathy. Class A represents a well-compensated disease, class B represents a significant functional compromise, and class C represents an advanced level of hepatic dysfunction or decompensated disease [8].

**Table 1:** Child-Pugh scoring system INR, international normalized ratio

Measure	1 point	2 points	3 points
Total bilirubin, mg/dL	<2	2-3	>3
Serum albumin, g/dL	>3.5	2.8-3.5	<2.8
INR	<1.7	1.7-2.3	>2.3
Ascites	None	Mild	Moderate
Hepatic encephalopathy	None	Grade I-II	Grade III-IV

Patients’ demographic and clinical information was recorded in standardized proformas after obtaining informed consent. Data were assessed using Statistical Package for the Social Sciences (SPSS) version 25 for Windows (IBM SPSS Statistics, Armonk, NY, USA). Categorical data were expressed as numbers and percentages, while quantitative variables were shown as mean and standard deviation. *Helicobacter pylori* infection was detected by testing stool antigens. Furthermore, the association of *H. pylori* with PHG in patients with and without cirrhosis was assessed using the chi-square test. A p-value of ≤0.05 was considered statistically significant. Multiple regression analysis was used to predict the association of portal hypertensive gastropathy with *H. pylori*, Child-Pugh score (liver disease severity), age, and gender.

## Results

The mean (±SD) age of the patients was 53.68 (±12.287) years, while the range was from 18 to 75 years. The majority of patients were male at 92/120 (77%), and the rest were females. A total of 84 patients had hepatitis C, nine patients had hepatitis B, including one patient with hepatitis B and C coinfection, while the rest of the 27 patients were negative for both hepatitis B and C. The baseline characteristics of the patients are shown in Table 2.

**Table 2:** Baseline and demographic features of the study population (n=120) INR, international normalized ratio; ALT, alanine aminotransferase

Total number of patients (N=120)	Mean	Standard deviation
Age (years)	53.68	12.3
INR (normal: ≤1.1)	1.298	0.3
Platelet count, ×10^9^/L (normal: 150-450×10^9^/L)	203.03	97.1
Albumin, g/dL (normal: 3.5-5.5 g/dL)	32.84	7.6
Hemoglobin, g/dL (normal: 11.6-15 g/dL)	11.94	2.4
Bilirubin, mg/dL (normal: 0.1-1.2 mg/dL)	18.52	11.9
ALT, U/L (normal: 4-36 U/L)	53.76	46.1

Out of the 40 patients who had normal liver but positive *H. pylori* stool antigen, only three (7.5%) had portal hypertensive gastropathy, which was mild. These patients underwent hepatic venous and arterial Doppler ultrasound scans. Two out of these three were found to have hepatic venous thrombosis, while the third one had normal hepatic and portal veins. Table 3 shows *H. pylori*, Child-Pugh class, and PHG in patients with and without cirrhosis, while Table 4 shows the association between Child-Pugh class and PHG. 

**Table 3:** Helicobacter pylori, Child-Pugh class, and portal hypertensive gastropathy in patients with and without cirrhosis *H. pylori*, *Helicobacter pylori*

H. pylori		With cirrhosis (Child-Pugh class)			Without cirrhosis	Portal hypertensive gastropathy			Total
		Class A	Class B	Class C		Normal	Mild	Severe	
	Negative	7	17	16	0	25	10	5	40
	Positive	17	9	14	40	51	28	1	80

**Table 4:** Association between Child-Pugh class and portal hypertensive gastropathy PHG, portal hypertensive gastropathy

	Normal	Mild PHG	Severe PHG	Total
Class A	17	7	0	24
Class B	13	13	0	26
Class C	10	14	6	30
No cirrhosis	36	4	0	40
Total	76	38	6	120

A chi-square test was used to find an association between *H. pylori* and portal hypertensive gastropathy in the overall study population. This showed a positive association, with a p-value of 0.022.

Of patients with liver cirrhosis who were *H. pylori*-negative, 37.5% (15/40) had portal hypertensive gastropathy. Of these, 12.5% (5/40) had severe PHG, while 25% (10/40) had mild PHG. Of patients with liver cirrhosis who were *H. pylori-*positive, 62.5% (25/40) had PHG. Of these, 2.5% (1/40) had severe PHG, while 60% (24/40) had mild PHG (Table 5).

**Table 5:** Association of H. pylori with PHG in overall patients *H. pylori*, *Helicobacter pylori*; PHG, portal hypertensive gastropathy

		Baveno PHG			Total	p-value
		Normal	Mild	Severe		
H. pylori	Negative	25	10	5	40	0.022
	Positive	51	28	1	80	
Total		76	38	6	120	

A subgroup analysis of patients with cirrhosis was carried out to see any possible association between *H. pylori* positivity and portal hypertensive gastropathy. It again showed a positive association, with a p-value of 0.004 (Table 6).

**Table 6:** Association of H. pylori with PHG in patients with cirrhosis *H. pylori*, *Helicobacter pylori*; PHG, portal hypertensive gastropathy

		Baveno PHG			Total	p-value
		Normal	Mild	Severe		
H. pylori	Negative	25	10	5	40	0.004
	Positive	15	24	1	40	
Total		40	34	6	80	

Since cirrhosis, even without H. pylori also cause portal hypertensive gastropathy, that's why a multiple regression analysis was done to predict the association of portal hypertensive gastropathy, with H. pylori, Child-Pugh class (severity of liver disease) age, and gender. Child-Pugh class predicted statistically significant portal hypertensive gastropathy, F (4,115)=6.522, p<0.0005. Others, which added statistical significance to the prediction included age, p=<0.0005, and gender (p= 0.016), while H. pylori added non-significantly, p=0.080.

## Discussion

Portal hypertension produces changes in gastric mucosa, including alteration in vascular caliber, structure, and amount of blood flow. *Helicobacter pylori*, on the other hand, also alters gastric and duodenal microcirculation [5,9].

The relationship between *H. pylori* and the development of portal hypertensive gastropathy in the presence of cirrhosis remains controversial. Numerous studies have shown an increased prevalence of *H. pylori* in portal hypertensive gastropathy; however, the true mechanism for this higher prevalence remains obscure, with different studies postulating different mechanisms. One of the popular theories is that *H. pylori* causes hypoxic mucosal surface and diminished epithelial integrity, which may facilitate colonization with this organism, while others claim that mucosal swelling and congestion, which increase nitric oxide, act as a suitable media for the growth of *H. pylori* [5-7,10]. Still, another famous theory is that in PHG, there is mucosal thinning and higher pH with decreased prostaglandin levels, facilitating *H. pylori*. Normal gastric pH is usually less than 4, while *H. pylori* thrives at a pH of about 6.1 [11].

Some studies have tried to relate PHG and *H. pylori* only by its presence. These studies have reported that patients with liver cirrhosis and portal hypertension have an increased prevalence of *H. pylori* as compared to the general population. This suggests the plausible role of *H. pylori* in the pathogenesis of PHG. Others have reported the effect of* H. pylori* on the severity of PHG by postulating that both liver cirrhosis and PHG are more severe in those with this infection as compared to those without it [5,12].

The study by Alarfaj et al. is unique as not only they observed a positive association in frequency and severity of PHG with *H. pylori*, but they also performed repeat endoscopy after the eradication of *H. pylori* with clarithromycin-based triple therapy. Endoscopy post-eradication showed significant improvement in PHG (p=0.045). However, there are various limitations of this study, as it neither elaborates on the time frame after the eradication of *H. pylori*, after which endoscopy was repeated, nor mentions whether patients received any other treatment for liver cirrhosis or PHG in the interim [5].

On the other hand, there are scores of studies that have not found any correlation between *H. pylori* and PHG and have reported that the prevalence of* H. pylori* in patients with PHG was indeed statistically insignificant [13-16]. Local literature from Southeast Asia showed that the severity of PHG did not correlate with the severity of liver disease as judged by the Child-Pugh score. In addition, the presence of *H. pylori* did not affect the severity of portal hypertensive gastropathy [13]. In our study, 62.5% of patients with cirrhosis who also had portal hypertensive gastropathy were *H. pylori-*positive, with a p-value of 0.004. The results are parallel to an Indian study, in which they performed *H. pylori* testing post-endoscopy in patients with cirrhosis and portal hypertension, as well as in those with cirrhosis without portal hypertension. They observed a significant association between *H. pylori* and PHG in patients with cirrhosis, with a p-value of 0.034. Not only was PHG more prevalent but also was severe in patients with cirrhosis who were *H. pylori-*positive. However, the drawback of this study was that it used *H. pylori* serology to diagnose *H. pylori*, which is not a very reliable method and can not differentiate between past and current infection [10].

The positive association between the severity of liver cirrhosis and the appearance of portal hypertensive gastropathy was shown in a study by Sarin et al. [17] and Sunkara et al. [18]. The study conducted by Sarin et al. differs from ours as they included sclerotherapy-treated patients. Out of our patients with cirrhosis, 24 were in class A, 26 were in class B, and 30 were in class C. If we analyze the outcomes related to the Child-Pugh score, out of 38 patients with mild PHG, four were without cirrhosis, while 34 were with cirrhosis. Out of these 34 patients, 14 were in class C, 13 were in class B, and seven were in class A. Six patients had severe PHG, and all of these were in class C. Thus, severe PHG was more often seen in patients with advanced liver disease (p-value = 0.029). Looking grossly, our results also depicted a positive association between *H. pylori* positivity and portal hypertensive gastropathy, with a p-value of 0.004.

We know that liver cirrhosis itself causes portal hypertension. We performed a multiple regression analysis. When confounding factors were controlled, including the severity of liver disease as assessed by Child-Pugh class, age, gender, and platelet count, no significant association was established between *H. pylori* and portal hypertensive gastropathy, signifying the fact that it is basically cirrhosis and not* H. pylori* that contributes to portal hypertension. Similar results were noted in a thoroughly done study on 140 patients with liver cirrhosis at Aga Khan Hospital in Karachi by Abbas et al., in which they assessed *H. pylori* by performing histology and polymerase chain reaction (PCR). They also studied virulence factors associated with the pathogenicity of *H. pylori* and compared mild or absent gastropathy with that of moderate or severe. In this benchmark study, they found no significant association between *H. pylori* and PHG [15]. Other studies have also shown no correlation between *H. pylori*, the stage of cirrhosis, and the presence and severity of PHG [19,20].

Our study has some limitations, including a small sample size, especially of patients without cirrhosis. The etiology of cirrhosis was not taken into account, and it was an unblinded study. Also, patients with *H. pylori* had already obtain stool antigen tests done in outpatient clinics, so due to financial constraints, a gastric biopsy, which is the gold standard for infection detection, was not performed. Prospective studies with larger sample sizes can be done to remove any ambiguities, if any.

## Conclusions

*Helicobacter pylori*-induced gastritis is an important cause of GI bleeding. Similarly, portal hypertensive gastropathy, mainly due to advanced liver disease, is another common and preventable cause of upper GI bleed. Both these conditions alter the mucosa of the stomach and can be the cause of serious GI bleed, which needs to be diagnosed and treated timely to prevent catastrophic complications.

Interlinking these diverse conditions appears an attractive idea from a clinician’s point of view, as theoretically, both these life-threatening conditions would be amenable to a single treatment: the eradication of *H. pylori*. Some of the previous studies had shown a positive association between these two clinical conditions, while to make matters more confusing, many rigorously done studies showed no relation at all. If we look at the results of our study, there does appear to be a positive significant association between *H. pylori* and PHG, if we apply only chi-square analysis, with a very significant p-value of 0.004. However, given the fact that both of these conditions are endemic in our area, it makes the association very likely. Controlling the confounding factors resolves the dilemma. That is, these conditions do not appear to have any significant statistical correlation. So, in brief, our study adds up to the other studies that show a negative association. Perhaps, it is time to move on to look for other causes of portal hypertensive gastropathy in liver cirrhosis beyond *H. pylori*.
